# Physician requirements for adoption of telehealth following the SARS-CoV-2 pandemic

**DOI:** 10.1038/s41746-021-00390-y

**Published:** 2021-02-09

**Authors:** Michael Hodgkins, Meg Barron, Shireesha Jevaji, Stacy Lloyd

**Affiliations:** American Medical Association, Chicago, IL USA

**Keywords:** Public health, Translational research

## Abstract

It took the advent of SARS-CoV-2, a “black swan event”, to widely introduce telehealth, remote care, and virtual house calls. Prior to the epidemic (2019), the American Medical Association (AMA) conducted a routine study to compare physicians’ adoption of emerging technologies to a similar survey in 2016. Most notable was a doubling in the adoption of telehealth/virtual technology to 28% and increases in the use of remote monitoring and management for improved care (13–22%). These results may now seem insignificant when compared to the unprecedented surge in telehealth visits because of SARS-CoV-2. Even as this surge levels off and begins to decline, many observers believe we will continue to see a persistent increase in the use of virtual visits compared to face-to-face care. The requirements for adoption communicated by physicians in both the 2016 and 2019 surveys are now more relevant than ever: Is remote care as effective as in-person care and how best to determine when to use these modalities? How do I safeguard my patients and my practice from liability and privacy concerns? How do I optimize using these technologies in my practice and, especially integration with my EHR and workflows to improve efficiency? And how will a mix of virtual and in-person visits affect practice revenue and sustainability? Consumers have also expressed concerns about payment for virtual visits as well as privacy and quality of care. If telehealth and remote care are here to stay, continuing to track their impact during the current public health emergency is critically important to address so that policymakers and insurers will take necessary steps to ensure that the “new normal” will reflect a health care delivery model that can provide comparable or improved results today and into the future.

## Introduction

In 2016, the AMA first benchmarked the integration of digital health tools into clinical practice. Among other findings, the research found that physicians believe telehealth tools can drive improved efficiency and safety in health care^1^.

Physicians surveyed in 2016 were optimistic about potential improvements to practice efficiencies and diagnostic abilities. Furthermore, physicians noted that liability coverage, data privacy, and workflow integration with electronic health record systems were important factors for digital health adoption^2^. A comparable survey conducted in 2019 demonstrated physician adoption of telehealth grew at a modest rate. Most notable was a doubling in the use of telehealth/virtual visit technology to 28% and increases in the use of remote monitoring and management for improved care (13–22%). However, these results may seem insignificant compared to the unprecedented surge in telehealth visits because of SARS-CoV-2, a “black swan” event of unprecedent proportion that has completely disrupted the care delivery model. An issue brief published July 2020 by Health and Human Services Assistant Secretary for Planning and Evaluation noted that nearly half (43.5%) of Medicare fee-for-service (FFS) primary care visits were provided via telehealth in April compared with less than one percent in February (0.1%) before a public health emergency was declared^3,4^. A survey of primary care and specialist practitioners conducted in April 2020 by IQVIA indicated a prepandemic rate of telehealth visits of 9% increasing to 51%. McKinsey has published similar results (11–46%) based on a consumer survey^5^.

Telehealth allows patients, especially those particularly vulnerable to SARS-CoV-19 complications, to access care safely. But the increase in telehealth visits and access to virtual services has not been without its challenges. A Health Affairs Blog notes that one in three U.S. households headed by those age 65 or older do not have computer access and more than half do not have a smartphone. Digital literacy is also an issue. Even with computer access, 52 million adults do not know how to use it properly, particularly among older and minority populations^6^. There are also notable differences associated with uneven access to broadband services in rural areas. While patients have noted positive factors associated with telehealth such as improved patient–clinician communication, and improved self-management^7^, they have also identified barriers that hindered their adoption of solutions including lack of validation, inability to use the technology, and lack of technical support^7^. They have expressed concerns about data privacy (24%), quality of care (64%), and losing a personal connection to their health care provider (45%)^8^. Additionally, patients reported interference with the patient–provider relationship to be a barrier to adoption^7^.

In a commentary on the shortfalls of telehealth, David Blumenthal points out the importance of a trusting relationship between patient and clinician and that clinicians rely on all their senses, not just hearing and vision. This is especially important when considering the complex patient with multiple conditions^9^. Hearing and vision cannot take the place of a blood pressure cuff, a spirometer or auscultation for evidence of a concerning murmur. Finally, the surge in virtual visits has not made up for the precipitous decline in face-to-face visits as evidenced by reports of a 55% drop in average practice revenue for predominately independent medical practices^10^. Practices also reported an increase in expenses associated with telehealth technology.

## The rise of the digital-native physician

The AMA survey results have provided deep insight into what physicians expect from digital technologies and a growing understanding of the key requirements for physicians to adopt new technology. There will soon be more ‘digitally-native’ physicians and other clinical professionals than those who have adapted to new technologies. This rise of the digitally native physician will profoundly impact health care delivery and patient outcomes, especially those seeking proven guidance and best practices needed to successfully integrate effective technology into practice^11^.

For this reason, the concerns expressed by physicians in both the 2016 and 2019 surveys are now more relevant than ever. Is remote care as effective as in-person care, and how best to determine when to use these modalities? How do I safeguard my patients and my practice from liability and privacy concerns? How do I optimize using these technologies in my practice and, especially integration with my EHR and workflows to improve efficiency? And how will a mix of virtual and in-person visits affect practice revenue and sustainability? The understandable push for telehealth use during the current epidemic has not allowed for a full airing of these concerns. Still, a growing body of evidence supports the need for careful consideration of how best to position telehealth in the future delivery system. Despite there still being some things to work out in telehealth services, one-fifth of physicians using video conferencing and telehealth tools expect to use them significantly more than before the pandemic^12^. Further exploration shows that physicians are interested in augmenting virtual care with additional digital health technologies, including remote patient monitoring, digital devices, and artificial intelligence, aligning with the 2019 survey results around emerging technology solutions^13^.

## Accelerating the growth of telehealth and remote care to meet needs

Even before SARS-CoV-2, it was already clear that telehealth was becoming a viable platform for physicians to provide clinical services. It provides physician practices, especially small practices with limited resources, a way to augment direct-to-consumer services currently offered and maintain continuity of care for their patients, which is a vital component of quality care.

Telehealth also has the potential to increase access to care and reduce health care costs. Regulatory and legislative changes and an increase in state telehealth parity laws could increase its prevalence. Still, the overall lack of data around its use to-date, financial impact, access to, and quality of care have historically been obstacles to adoption. Additional research is needed to provide evidence-based guidance to physicians on implementing telehealth in their practices.

SARS-CoV-2 has made telehealth an essential tool to protect access to care and to respond to public health needs. State requirements for nonessential workers to stay at home and easing federal and state restrictions during the pandemic have exponentially increased telehealth use to screen patients for the virus and deliver other essential medical care. Some have observed that patients’ convenience and the demonstrated ability to deliver routine care using telehealth will lead to a “new normal” for physicians and patients. Anecdotal patient reports and satisfaction scores share preliminary findings on improved satisfaction, access to care and timeliness of care, serving as indicators of quality, efficient care. But the public health emergency has also emphasized the need to address concerns around the lack of access to technology and high-speed internet, and low digital literacy that may prevent some populations from receiving care virtually.

Physicians’ opinions remain mixed about the future of telehealth in their practices. Anecdotal reports point to physician concerns over interference in their ongoing relationship with patients, which is an essential component of delivering high-quality care. Integration of telehealth technology with the EHR and practice workflows, so that care is properly documented, are additional challenges. The future of reimbursement for the use of telehealth remains unclear as does the right case mix of virtual and face-to-face visits. During the pandemic, states and the federal government implemented temporary emergency measures that relaxed many existing telehealth regulations. These new provisions included the use of technologies that do not provide sufficient privacy protections. Many physicians have uncertainty around the scalability of telehealth in routine practice once the emergency mandates have ended. While the 2019 survey results indicated that physicians are increasingly looking to integrate telehealth and mobile health technologies into physician practices so that they may understand and manage patients with chronic diseases outside of the practice environment, the unprecedented increase in telehealth use during the current crisis is both a challenge and an opportunity to assess its impact on access to care, the quality of care, and the financial impact on the health care system.

## Integrating the physician perspective into telehealth technology

Challenges around usability and workflow integration still exist today with EHRs. With lessons learned from the lack of physician input in the development of these technologies and the resulting frustrations that have followed, it is critically important that a diverse group of industry, clinical, and technology partners continue to collaborate to integrate physician perspective to develop and scale the adoption of telehealth solutions that benefit both patients and clinicians. Through these connections, the industry continues to learn how to engage key stakeholders to drive successful health care innovation^8^.

Recognizing the need for collaboration to address these and other barriers to adoption, various stakeholders have previously assembled and identified the need for an industry roadmap to efficiently and effectively implement digital health solutions in practice, including telehealth and remote patient monitoring^5^. Resources are available that aggregate key steps, best practices and common challenges, and provide case studies to help physicians and practices navigate and maximize technology for improved patient care and professional satisfaction^6^. The recent surge in virtual health care services opens the door to better understand physician and patient experiences with these tools and develop more refined best practices for sustainable use.

## Leading the charge in expediting telehealth implementation into practice

With many state and local authorities issuing quarantine-in-place orders across the country, physicians naturally turned to telehealth to keep communication lines and care open with their patients. Amid the pandemic, the AMA has been a leading physician and patient ally—voicing recommendations to key Congressional leaders and agency staff, state policymakers, and private sector stakeholders, emphasizing the requirements for adoption raised by physicians in both the 2016 and 2019 AMA surveys. This effort included working with private insurers to help physicians navigate the myriad of private payment requirements for telehealth services^14,15^ and provide guidance for physicians to implement and adapt telehealth programs to continue care quickly. Notably, AMA research spurred solutions that keep physician and patient needs at the forefront of telehealth delivery. The Telehealth Initiative—a collaboration between the AMA, The Physicians Foundation, Florida Medical Association, Massachusetts Medical Society and Texas Medical Association, supports physicians as they embrace new telehealth care delivery models.

In terms of addressing the regulatory and health equity challenges ahead, the AMA is supporting S. 4375, the “Telehealth Modernization Act of 2020,” which would permanently remove many of the regulatory restrictions on telehealth that were temporarily lifted at the start of the pandemic and have enabled physicians to deliver care to patients more readily. AMA is committed to addressing the longstanding health inequities among marginalized and communities impacted disproportionately by SARS-CoV-19, and is working to identify the physician’s role in dismantling patient barriers to accessing care.

Additionally, the AMA recently adopted new telehealth policy including measures to ensure the continuity of telehealth adoption after the pandemic; the adoption of uniform laws and regulations by the federal and state governments and the payer industry to ensure privacy and security standards are upheld; and that telehealth services provide equitable coverage and access especially for at-risk and underserved populations to reduce health disparities^16^.

While implementing these policies is critical, further research is needed to understand which patient populations are best suited for telehealth services, finding the right balance between virtual and in-person care and long-term implications for the delivery system. Specifically, clinical outcomes, cost of care and both patient and physician satisfaction.

## Conclusion

As we continue to consider physician requirements for care delivery, virtual access will be a necessity. While the new baseline for telehealth use is still unclear, research has shown that the adoption of digital tools into clinical practice was already growing.

Physicians are attracted to evidence-based technologies and digital tools that increase patient safety, convenience, and adherence, in addition to improving clinical workflows and providing decision support^2^. Improved efficiency and increased patient safety were among the primary drivers in physician adoption of digital solutions^2,4,15^. The advent of SARS-CoV-2 spurred an exponential increase in telehealth use and has created a heightened interest in how digital solutions can impact care but many questions remain unanswered. One should view the current crisis as both a challenge and an opportunity to assess the impact of digital tools on access to care, the quality of care, and the financial impact on the health care ecosystem.
